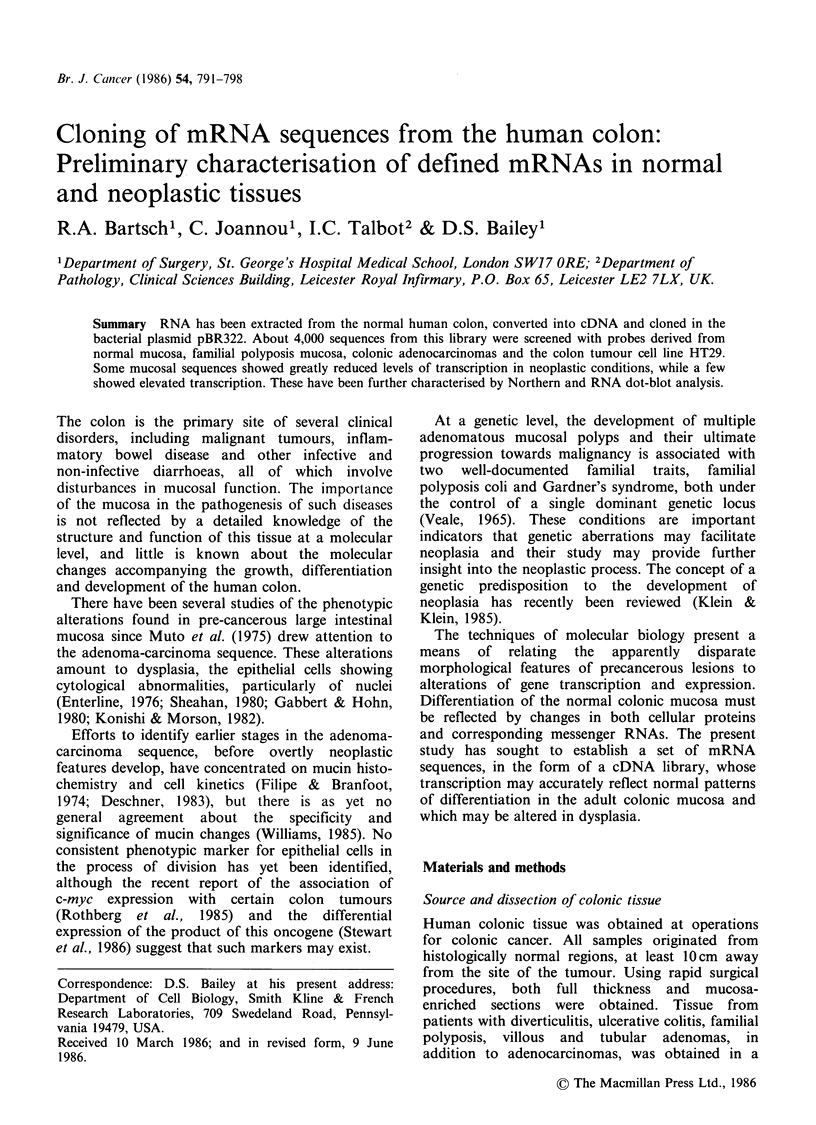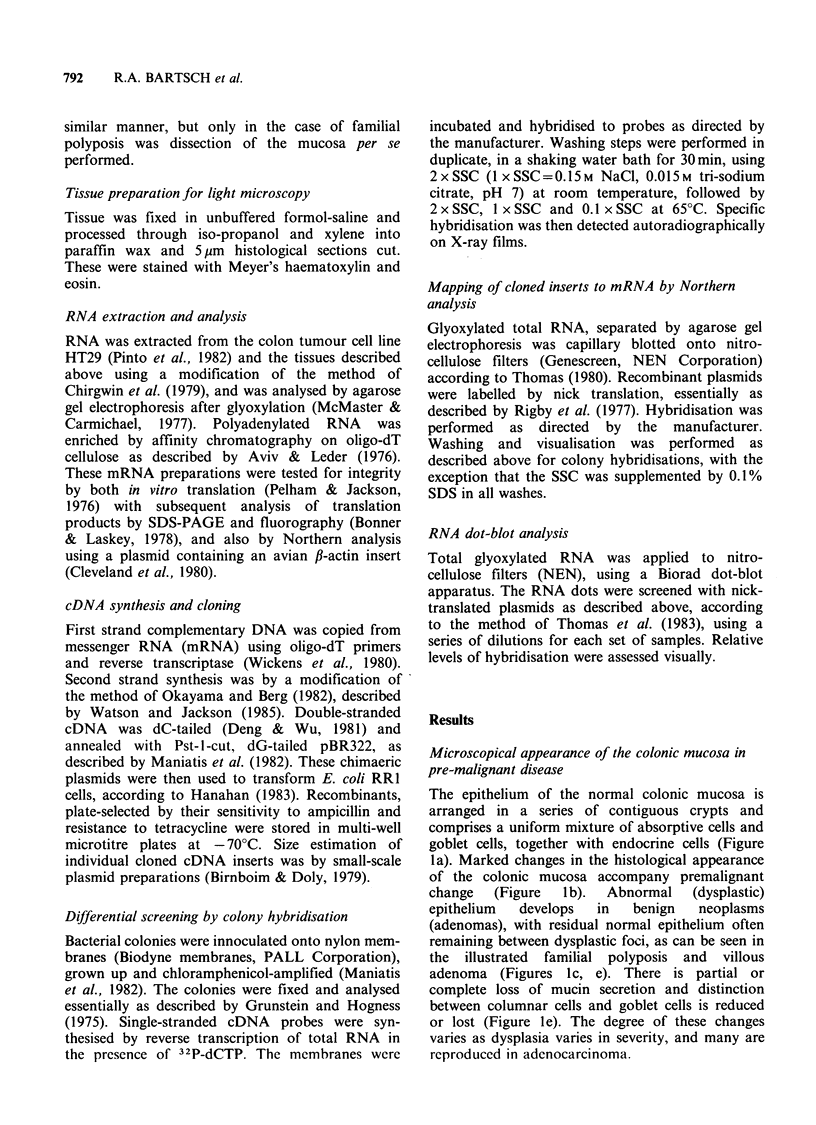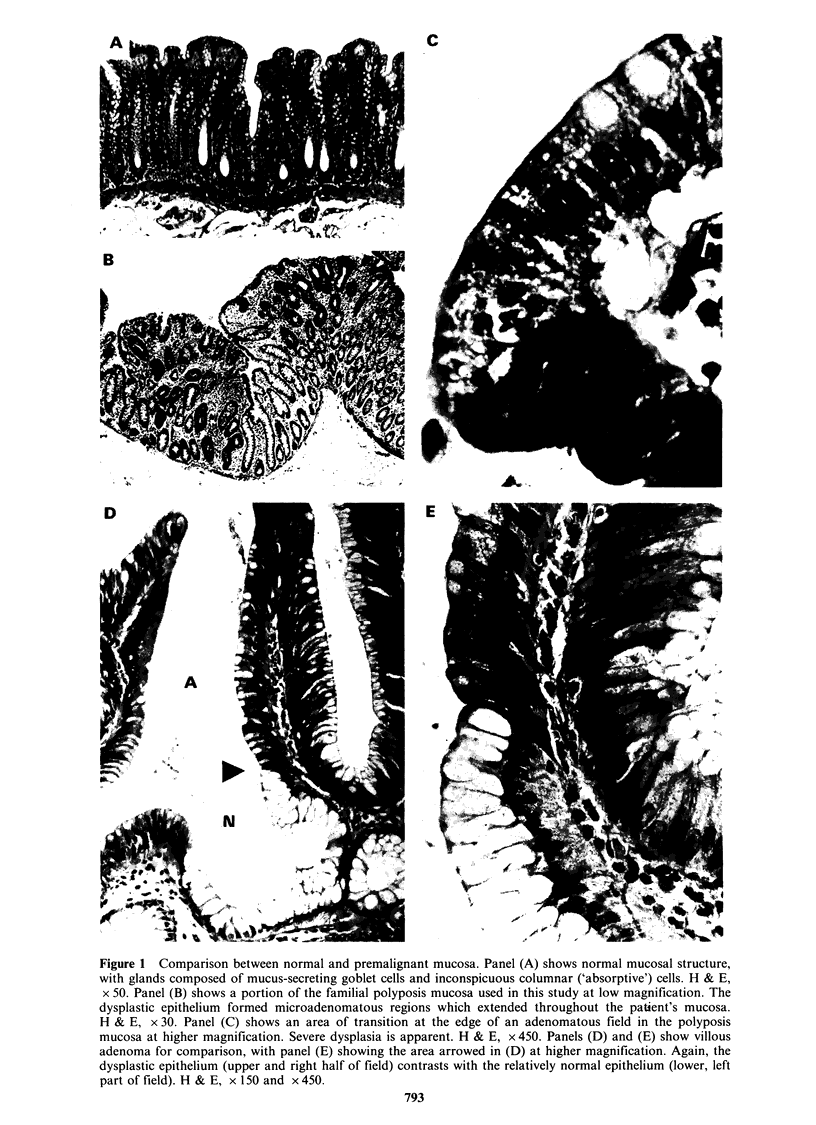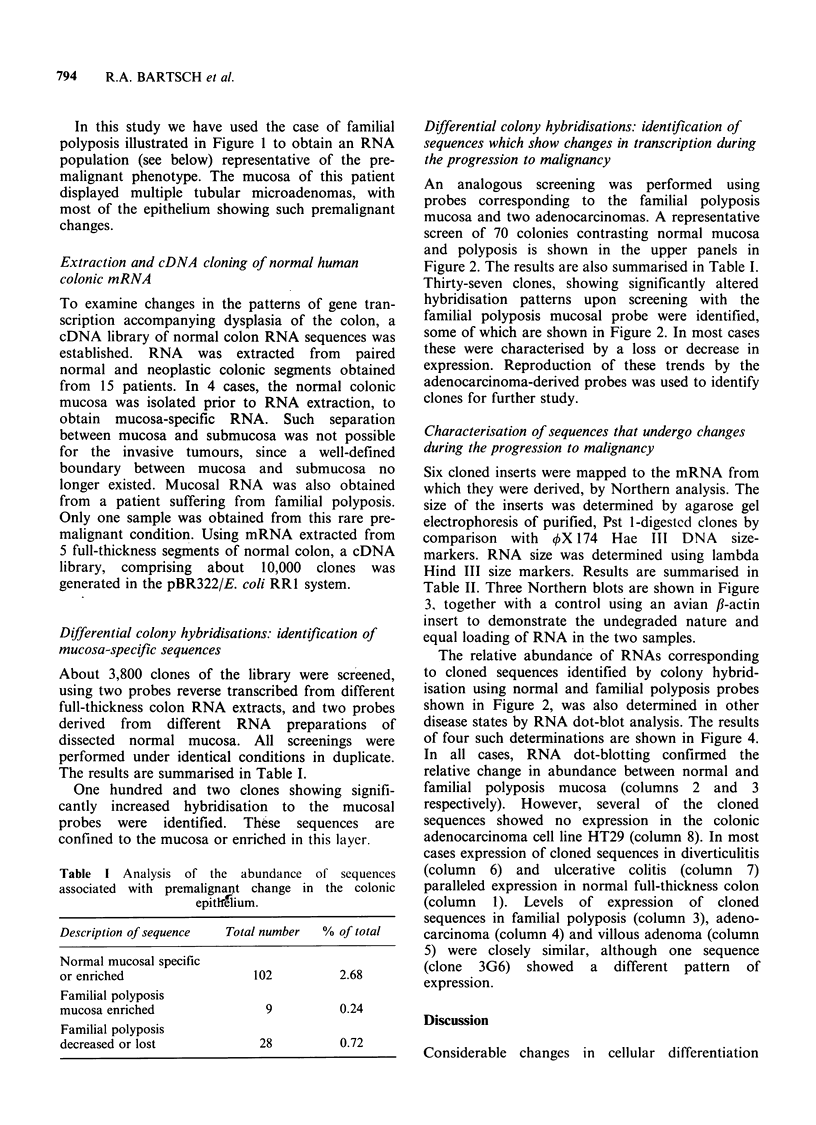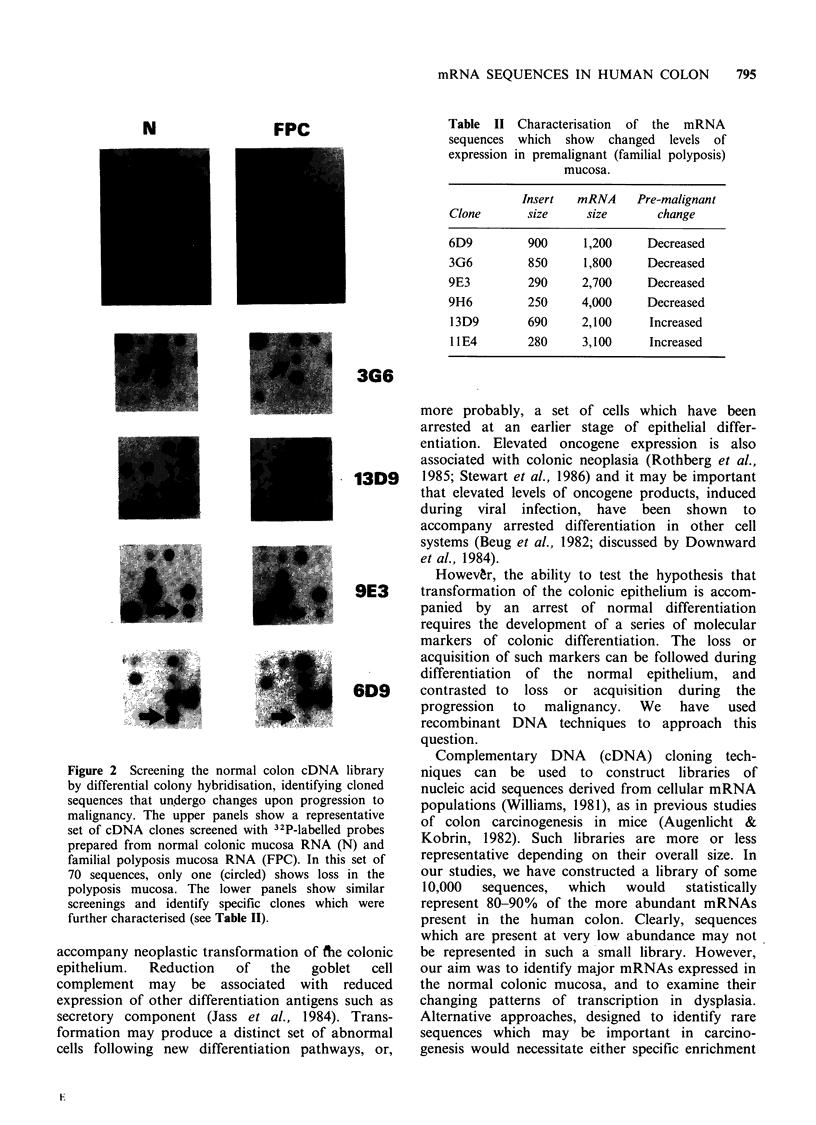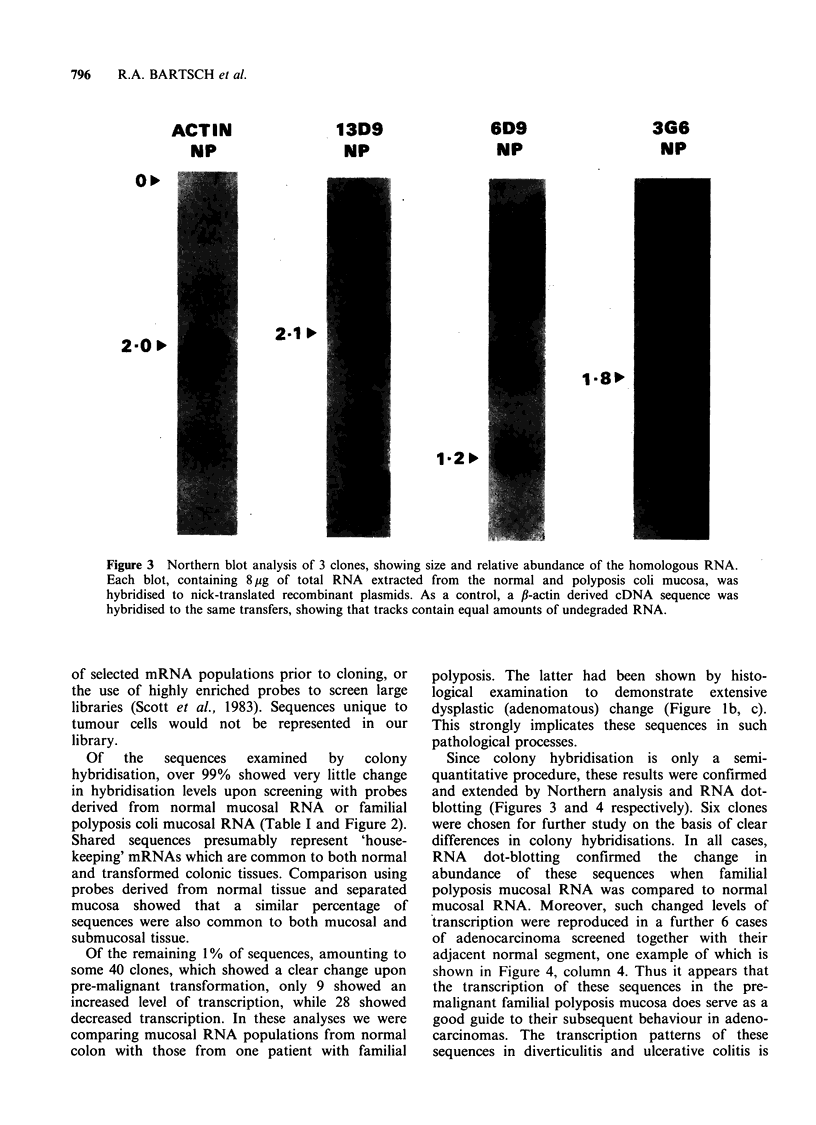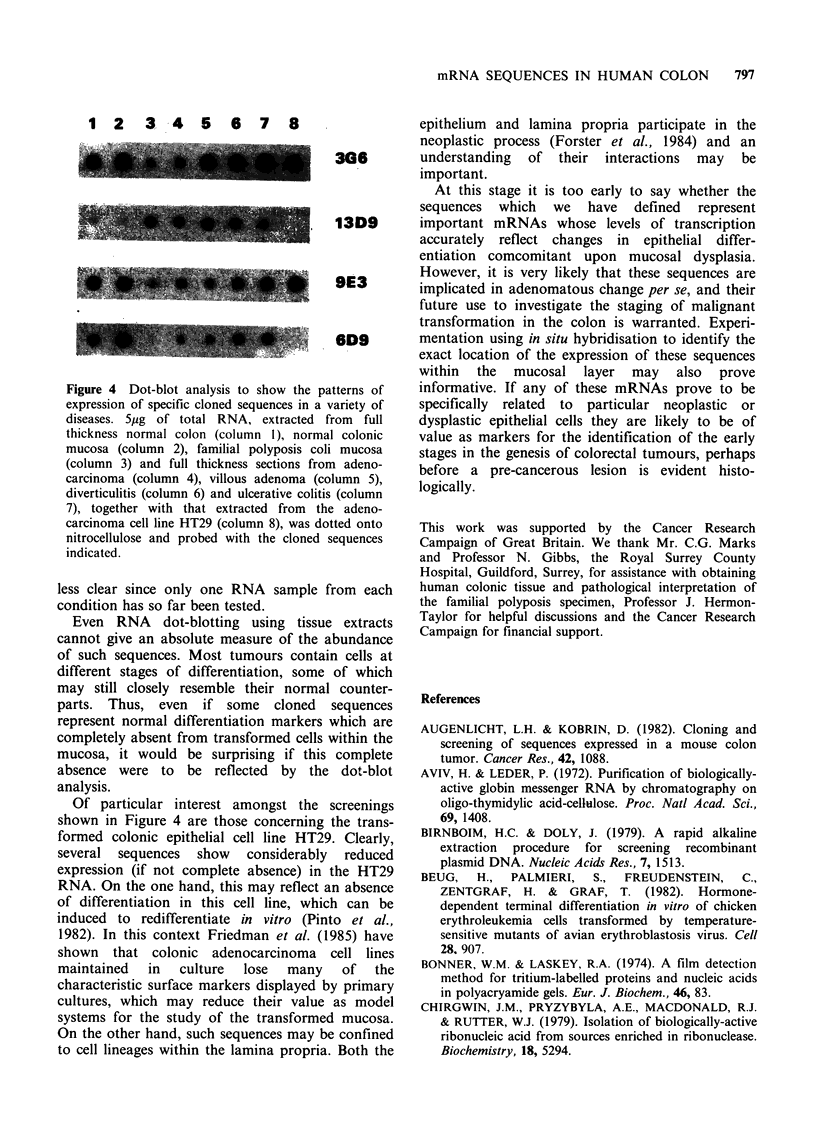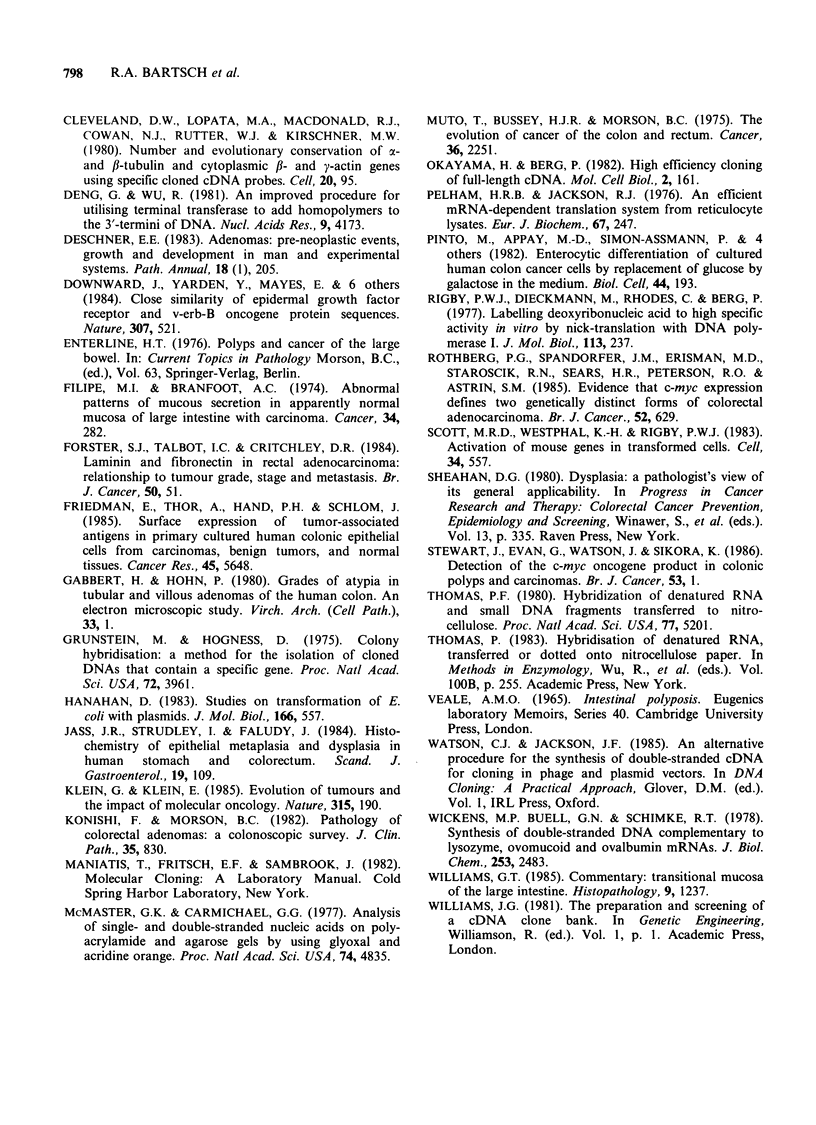# Cloning of mRNA sequences from the human colon: preliminary characterisation of defined mRNAs in normal and neoplastic tissues.

**DOI:** 10.1038/bjc.1986.242

**Published:** 1986-11

**Authors:** R. A. Bartsch, C. Joannou, I. C. Talbot, D. S. Bailey

## Abstract

**Images:**


*Br. J. Cancer* (1986) **54**, 791–798

### Cloning of mRNA sequences from the human colon: Preliminary characterisation of defined mRNAs in normal and neoplastic tissues

R.A. Bartsch[1], C. Joannou[1], I.C. Talbot[2] & D.S. Bailey[1]

[1]*Department of Surgery, St. George's Hospital Medical School, London SW17 0RE;* [2]*Department of Pathology, Clinical Sciences Building, Leicester Royal Infirmary, P.O. Box 65, Leicester LE2 7LX, UK.*

**Summary** RNA has been extracted from the normal human colon, converted into cDNA and cloned in the bacterial plasmid pBR322. About 4,000 sequences from this library were screened with probes derived from normal mucosa, familial polyposis mucosa, colonic adenocarcinomas and the colon tumour cell line HT29. Some mucosal sequences showed greatly reduced levels of transcription in neoplastic conditions, while a few showed elevated transcription. These have been further characterised by Northern and RNA dot-blot analysis.

The colon is the primary site of several clinical disorders, including malignant tumours, inflammatory bowel disease and other infective and non-infective diarrhoeas, all of which involve disturbances in mucosal function. The importance of the mucosa in the pathogenesis of such diseases is not reflected by a detailed knowledge of the structure and function of this tissue at a molecular level, and little is known about the molecular changes accompanying the growth, differentiation and development of the human colon.

There have been several studies of the phenotypic alterations found in pre-cancerous large intestinal mucosa since Muto *et al.* (1975) drew attention to the adenoma-carcinoma sequence. These alterations amount to dysplasia, the epithelial cells showing cytological abnormalities, particularly of nuclei (Enterline, 1976; Sheahan, 1980; Gabbert & Hohn, 1980; Konishi & Morson, 1982).

Efforts to identify earlier stages in the adenoma-carcinoma sequence, before overtly neoplastic features develop, have concentrated on mucin histochemistry and cell kinetics (Filipe & Branfoot, 1974; Deschner, 1983), but there is as yet no general agreement about the specificity and significance of mucin changes (Williams, 1985). No consistent phenotypic marker for epithelial cells in the process of division has yet been identified, although the recent report of the association of c-*myc* expression with certain colon tumours (Rothberg *et al.*, 1985) and the differential expression of the product of this oncogene (Stewart *et al.*, 1986) suggest that such markers may exist.

Correspondence: D.S. Bailey at his present address: Department of Cell Biology, Smith Kline & French Research Laboratories, 709 Swedeland Road, Pennsylvania 19479, USA.

Received 10 March 1986; and in revised form, 9 June 1986.

At a genetic level, the development of multiple adenomatous mucosal polyps and their ultimate progression towards malignancy is associated with two well-documented familial traits, familial polyposis coli and Gardner's syndrome, both under the control of a single dominant genetic locus (Veale, 1965). These conditions are important indicators that genetic aberrations may facilitate neoplasia and their study may provide further insight into the neoplastic process. The concept of a genetic predisposition to the development of neoplasia has recently been reviewed (Klein & Klein, 1985).

The techniques of molecular biology present a means of relating the apparently disparate morphological features of precancerous lesions to alterations of gene transcription and expression. Differentiation of the normal colonic mucosa must be reflected by changes in both cellular proteins and corresponding messenger RNAs. The present study has sought to establish a set of mRNA sequences, in the form of a cDNA library, whose transcription may accurately reflect normal patterns of differentiation in the adult colonic mucosa and which may be altered in dysplasia.

### Materials and methods

### *Source and dissection of colonic tissue*

Human colonic tissue was obtained at operations for colonic cancer. All samples originated from histologically normal regions, at least 10 cm away from the site of the tumour. Using rapid surgical procedures, both full thickness and mucosa-enriched sections were obtained. Tissue from patients with diverticulitis, ulcerative colitis, familial polyposis, villous and tubular adenomas, in addition to adenocarcinomas, was obtained in a

© The Macmillan Press Ltd., 1986

similar manner, but only in the case of familial polyposis was dissection of the mucosa *per se* performed.

*Tissue preparation for light microscopy*

Tissue was fixed in unbuffered formol-saline and processed through iso-propanol and xylene into paraffin wax and 5 $\mu$m histological sections cut. These were stained with Meyer's haematoxylin and eosin.

*RNA extraction and analysis*

RNA was extracted from the colon tumour cell line HT29 (Pinto *et al.*, 1982) and the tissues described above using a modification of the method of Chirgwin *et al.* (1979), and was analysed by agarose gel electrophoresis after glyoxylation (McMaster & Carmichael, 1977). Polyadenylated RNA was enriched by affinity chromatography on oligo-dT cellulose as described by Aviv & Leder (1976). These mRNA preparations were tested for integrity by both *in vitro* translation (Pelham & Jackson, 1976) with subsequent analysis of translation products by SDS-PAGE and fluorography (Bonner & Laskey, 1978), and also by Northern analysis using a plasmid containing an avian $\beta$-actin insert (Cleveland *et al.*, 1980).

*cDNA synthesis and cloning*

First strand complementary DNA was copied from messenger RNA (mRNA) using oligo-dT primers and reverse transcriptase (Wickens *et al.*, 1980). Second strand synthesis was by a modification of the method of Okayama and Berg (1982), described by Watson and Jackson (1985). Double-stranded cDNA was dC-tailed (Deng & Wu, 1981) and annealed with Pst-1-cut, dG-tailed pBR322, as described by Maniatis *et al.* (1982). These chimaeric plasmids were then used to transform *E. coli* RR1 cells, according to Hanahan (1983). Recombinants, plate-selected by their sensitivity to ampicillin and resistance to tetracycline were stored in multi-well microtitre plates at $-70$°C. Size estimation of individual cloned cDNA inserts was by small-scale plasmid preparations (Birnboim & Doly, 1979).

*Differential screening by colony hybridisation*

Bacterial colonies were innoculated onto nylon membranes (Biodyne membranes, PALL Corporation), grown up and chloramphenicol-amplified (Maniatis *et al.*, 1982). The colonies were fixed and analysed essentially as described by Grunstein and Hogness (1975). Single-stranded cDNA probes were synthesised by reverse transcription of total RNA in the presence of $^{32}$P-dCTP. The membranes were incubated and hybridised to probes as directed by the manufacturer. Washing steps were performed in duplicate, in a shaking water bath for 30 min, using 2 × SSC (1 × SSC = 0.15 M NaCl, 0.015 M tri-sodium citrate, pH 7) at room temperature, followed by 2 × SSC, 1 × SSC and 0.1 × SSC at 65°C. Specific hybridisation was then detected autoradiographically on X-ray films.

*Mapping of cloned inserts to mRNA by Northern analysis*

Glyoxylated total RNA, separated by agarose gel electrophoresis was capillary blotted onto nitrocellulose filters (Genescreen, NEN Corporation) according to Thomas (1980). Recombinant plasmids were labelled by nick translation, essentially as described by Rigby *et al.* (1977). Hybridisation was performed as directed by the manufacturer. Washing and visualisation was performed as described above for colony hybridisations, with the exception that the SSC was supplemented by 0.1% SDS in all washes.

*RNA dot-blot analysis*

Total glyoxylated RNA was applied to nitrocellulose filters (NEN), using a Biorad dot-blot apparatus. The RNA dots were screened with nick-translated plasmids as described above, according to the method of Thomas *et al.* (1983), using a series of dilutions for each set of samples. Relative levels of hybridisation were assessed visually.

### Results

*Microscopical appearance of the colonic mucosa in pre-malignant disease*

The epithelium of the normal colonic mucosa is arranged in a series of contiguous crypts and comprises a uniform mixture of absorptive cells and goblet cells, together with endocrine cells (Figure 1a). Marked changes in the histological appearance of the colonic mucosa accompany premalignant change (Figure 1b). Abnormal (dysplastic) epithelium develops in benign neoplasms (adenomas), with residual normal epithelium often remaining between dysplastic foci, as can be seen in the illustrated familial polyposis and villous adenoma (Figures 1c, e). There is partial or complete loss of mucin secretion and distinction between columnar cells and goblet cells is reduced or lost (Figure 1e). The degree of these changes varies as dysplasia varies in severity, and many are reproduced in adenocarcinoma.

**Figure 1** Comparison between normal and premalignant mucosa. Panel (A) shows normal mucosal structure, with glands composed of mucus-secreting goblet cells and inconspicuous columnar ('absorptive') cells. H & E, ×50. Panel (B) shows a portion of the familial polyposis mucosa used in this study at low magnification. The dysplastic epithelium formed microadenomatous regions which extended throughout the patient's mucosa. H & E, ×30. Panel (C) shows an area of transition at the edge of an adenomatous field in the polyposis mucosa at higher magnification. Severe dysplasia is apparent. H & E, ×450. Panels (D) and (E) show villous adenoma for comparison, with panel (E) showing the area arrowed in (D) at higher magnification. Again, the dysplastic epithelium (upper and right half of field) contrasts with the relatively normal epithelium (lower, left part of field). H & E, ×150 and ×450.

In this study we have used the case of familial polyposis illustrated in Figure 1 to obtain an RNA population (see below) representative of the premalignant phenotype. The mucosa of this patient displayed multiple tubular microadenomas, with most of the epithelium showing such premalignant changes.

*Extraction and cDNA cloning of normal human colonic mRNA*

To examine changes in the patterns of gene transcription accompanying dysplasia of the colon, a cDNA library of normal colon RNA sequences was established. RNA was extracted from paired normal and neoplastic colonic segments obtained from 15 patients. In 4 cases, the normal colonic mucosa was isolated prior to RNA extraction, to obtain mucosa-specific RNA. Such separation between mucosa and submucosa was not possible for the invasive tumours, since a well-defined boundary between mucosa and submucosa no longer existed. Mucosal RNA was also obtained from a patient suffering from familial polyposis. Only one sample was obtained from this rare premalignant condition. Using mRNA extracted from 5 full-thickness segments of normal colon, a cDNA library, comprising about 10,000 clones was generated in the pBR322/*E. coli* RR1 system.

*Differential colony hybridisations: identification of mucosa-specific sequences*

About 3,800 clones of the library were screened, using two probes reverse transcribed from different full-thickness colon RNA extracts, and two probes derived from different RNA preparations of dissected normal mucosa. All screenings were performed under identical conditions in duplicate. The results are summarised in Table I.

One hundred and two clones showing significantly increased hybridisation to the mucosal probes were identified. These sequences are confined to the mucosa or enriched in this layer.

**Table I** Analysis of the abundance of sequences associated with premalignant change in the colonic epithelium.

| *Description of sequence* | *Total number* | *% of total* |
|---|---|---|
| Normal mucosal specific or enriched | 102 | 2.68 |
| Familial polyposis mucosa enriched | 9 | 0.24 |
| Familial polyposis decreased or lost | 28 | 0.72 |

*Differential colony hybridisations: identification of sequences which show changes in transcription during the progression to malignancy*

An analogous screening was performed using probes corresponding to the familial polyposis mucosa and two adenocarcinomas. A representative screen of 70 colonies contrasting normal mucosa and polyposis is shown in the upper panels in Figure 2. The results are also summarised in Table I. Thirty-seven clones, showing significantly altered hybridisation patterns upon screening with the familial polyposis mucosal probe were identified, some of which are shown in Figure 2. In most cases these were characterised by a loss or decrease in expression. Reproduction of these trends by the adenocarcinoma-derived probes was used to identify clones for further study.

*Characterisation of sequences that undergo changes during the progression to malignancy*

Six cloned inserts were mapped to the mRNA from which they were derived, by Northern analysis. The size of the inserts was determined by agarose gel electrophoresis of purified, Pst 1-digested clones by comparison with $\phi$X 174 Hae III DNA size-markers. RNA size was determined using lambda Hind III size markers. Results are summarised in Table II. Three Northern blots are shown in Figure 3, together with a control using an avian $\beta$-actin insert to demonstrate the undegraded nature and equal loading of RNA in the two samples.

The relative abundance of RNAs corresponding to cloned sequences identified by colony hybridisation using normal and familial polyposis probes shown in Figure 2, was also determined in other disease states by RNA dot-blot analysis. The results of four such determinations are shown in Figure 4. In all cases, RNA dot-blotting confirmed the relative change in abundance between normal and familial polyposis mucosa (columns 2 and 3 respectively). However, several of the cloned sequences showed no expression in the colonic adenocarcinoma cell line HT29 (column 8). In most cases expression of cloned sequences in diverticulitis (column 6) and ulcerative colitis (column 7) paralleled expression in normal full-thickness colon (column 1). Levels of expression of cloned sequences in familial polyposis (column 3), adenocarcinoma (column 4) and villous adenoma (column 5) were closely similar, although one sequence (clone 3G6) showed a different pattern of expression.

### Discussion

Considerable changes in cellular differentiation

**Figure 2** Screening the normal colon cDNA library by differential colony hybridisation, identifying cloned sequences that undergo changes upon progression to malignancy. The upper panels show a representative set of cDNA clones screened with $^{32}P$-labelled probes prepared from normal colonic mucosa RNA (N) and familial polyposis mucosa RNA (FPC). In this set of 70 sequences, only one (circled) shows loss in the polyposis mucosa. The lower panels show similar screenings and identify specific clones which were further characterised (see **Table II**).

accompany neoplastic transformation of the colonic epithelium. Reduction of the goblet cell complement may be associated with reduced expression of other differentiation antigens such as secretory component (Jass *et al.*, 1984). Transformation may produce a distinct set of abnormal cells following new differentiation pathways, or,

**Table II** Characterisation of the mRNA sequences which show changed levels of expression in premalignant (familial polyposis) mucosa.

| *Clone* | *Insert size* | *mRNA size* | *Pre-malignant change* |
|---|---|---|---|
| 6D9 | 900 | 1,200 | Decreased |
| 3G6 | 850 | 1,800 | Decreased |
| 9E3 | 290 | 2,700 | Decreased |
| 9H6 | 250 | 4,000 | Decreased |
| 13D9 | 690 | 2,100 | Increased |
| 11E4 | 280 | 3,100 | Increased |

more probably, a set of cells which have been arrested at an earlier stage of epithelial differentiation. Elevated oncogene expression is also associated with colonic neoplasia (Rothberg *et al.*, 1985; Stewart *et al.*, 1986) and it may be important that elevated levels of oncogene products, induced during viral infection, have been shown to accompany arrested differentiation in other cell systems (Beug *et al.*, 1982; discussed by Downward *et al.*, 1984).

However, the ability to test the hypothesis that transformation of the colonic epithelium is accompanied by an arrest of normal differentiation requires the development of a series of molecular markers of colonic differentiation. The loss or acquisition of such markers can be followed during differentiation of the normal epithelium, and contrasted to loss or acquisition during the progression to malignancy. We have used recombinant DNA techniques to approach this question.

Complementary DNA (cDNA) cloning techniques can be used to construct libraries of nucleic acid sequences derived from cellular mRNA populations (Williams, 1981), as in previous studies of colon carcinogenesis in mice (Augenlicht & Kobrin, 1982). Such libraries are more or less representative depending on their overall size. In our studies, we have constructed a library of some 10,000 sequences, which would statistically represent 80–90% of the more abundant mRNAs present in the human colon. Clearly, sequences which are present at very low abundance may not be represented in such a small library. However, our aim was to identify major mRNAs expressed in the normal colonic mucosa, and to examine their changing patterns of transcription in dysplasia. Alternative approaches, designed to identify rare sequences which may be important in carcinogenesis would necessitate either specific enrichment

**Figure 3** Northern blot analysis of 3 clones, showing size and relative abundance of the homologous RNA. Each blot, containing 8 $\mu$g of total RNA extracted from the normal and polyposis coli mucosa, was hybridised to nick-translated recombinant plasmids. As a control, a $\beta$-actin derived cDNA sequence was hybridised to the same transfers, showing that tracks contain equal amounts of undegraded RNA.

of selected mRNA populations prior to cloning, or the use of highly enriched probes to screen large libraries (Scott *et al.*, 1983). Sequences unique to tumour cells would not be represented in our library.

Of the sequences examined by colony hybridisation, over 99% showed very little change in hybridisation levels upon screening with probes derived from normal mucosal RNA or familial polyposis coli mucosal RNA (Table I and Figure 2). Shared sequences presumably represent 'housekeeping' mRNAs which are common to both normal and transformed colonic tissues. Comparison using probes derived from normal tissue and separated mucosa showed that a similar percentage of sequences were also common to both mucosal and submucosal tissue.

Of the remaining 1% of sequences, amounting to some 40 clones, which showed a clear change upon pre-malignant transformation, only 9 showed an increased level of transcription, while 28 showed decreased transcription. In these analyses we were comparing mucosal RNA populations from normal colon with those from one patient with familial polyposis. The latter had been shown by histological examination to demonstrate extensive dysplastic (adenomatous) change (Figure 1b, c). This strongly implicates these sequences in such pathological processes.

Since colony hybridisation is only a semi-quantitative procedure, these results were confirmed and extended by Northern analysis and RNA dot-blotting (Figures 3 and 4 respectively). Six clones were chosen for further study on the basis of clear differences in colony hybridisations. In all cases, RNA dot-blotting confirmed the change in abundance of these sequences when familial polyposis mucosal RNA was compared to normal mucosal RNA. Moreover, such changed levels of transcription were reproduced in a further 6 cases of adenocarcinoma screened together with their adjacent normal segment, one example of which is shown in Figure 4, column 4. Thus it appears that the transcription of these sequences in the pre-malignant familial polyposis mucosa does serve as a good guide to their subsequent behaviour in adenocarcinomas. The transcription patterns of these sequences in diverticulitis and ulcerative colitis is

**Figure 4** Dot-blot analysis to show the patterns of expression of specific cloned sequences in a variety of diseases. 5μg of total RNA, extracted from full thickness normal colon (column 1), normal colonic mucosa (column 2), familial polyposis coli mucosa (column 3) and full thickness sections from adeno-carcinoma (column 4), villous adenoma (column 5), diverticulitis (column 6) and ulcerative colitis (column 7), together with that extracted from the adeno-carcinoma cell line HT29 (column 8), was dotted onto nitrocellulose and probed with the cloned sequences indicated.

less clear since only one RNA sample from each condition has so far been tested.

Even RNA dot-blotting using tissue extracts cannot give an absolute measure of the abundance of such sequences. Most tumours contain cells at different stages of differentiation, some of which may still closely resemble their normal counter-parts. Thus, even if some cloned sequences represent normal differentiation markers which are completely absent from transformed cells within the mucosa, it would be surprising if this complete absence were to be reflected by the dot-blot analysis.

Of particular interest amongst the screenings shown in Figure 4 are those concerning the trans-formed colonic epithelial cell line HT29. Clearly, several sequences show considerably reduced expression (if not complete absence) in the HT29 RNA. On the one hand, this may reflect an absence of differentiation in this cell line, which can be induced to redifferentiate *in vitro* (Pinto *et al.*, 1982). In this context Friedman *et al.* (1985) have shown that colonic adenocarcinoma cell lines maintained in culture lose many of the characteristic surface markers displayed by primary cultures, which may reduce their value as model systems for the study of the transformed mucosa. On the other hand, such sequences may be confined to cell lineages within the lamina propria. Both the epithelium and lamina propria participate in the neoplastic process (Forster *et al.*, 1984) and an understanding of their interactions may be important.

At this stage it is too early to say whether the sequences which we have defined represent important mRNAs whose levels of transcription accurately reflect changes in epithelial differ-entiation comcomitant upon mucosal dysplasia. However, it is very likely that these sequences are implicated in adenomatous change *per se*, and their future use to investigate the staging of malignant transformation in the colon is warranted. Experi-mentation using *in situ* hybridisation to identify the exact location of the expression of these sequences within the mucosal layer may also prove informative. If any of these mRNAs prove to be specifically related to particular neoplastic or dysplastic epithelial cells they are likely to be of value as markers for the identification of the early stages in the genesis of colorectal tumours, perhaps before a pre-cancerous lesion is evident histo-logically.

This work was supported by the Cancer Research Campaign of Great Britain. We thank Mr. C.G. Marks and Professor N. Gibbs, the Royal Surrey County Hospital, Guildford, Surrey, for assistance with obtaining human colonic tissue and pathological interpretation of the familial polyposis specimen, Professor J. Hermon-Taylor for helpful discussions and the Cancer Research Campaign for financial support.

### References

AUGENLICHT, L.H. & KOBRIN, D. (1982). Cloning and screening of sequences expressed in a mouse colon tumor. *Cancer Res.*, **42**, 1088.

AVIV, H. & LEDER, P. (1972). Purification of biologically-active globin messenger RNA by chromatography on oligo-thymidylic acid-cellulose. *Proc. Natl Acad. Sci.*, **69**, 1408.

BIRNBOIM, H.C. & DOLY, J. (1979). A rapid alkaline extraction procedure for screening recombinant plasmid DNA. *Nucleic Acids Res.*, **7**, 1513.

BEUG, H., PALMIERI, S., FREUDENSTEIN, C., ZENTGRAF, H. & GRAF, T. (1982). Hormone-dependent terminal differentiation *in vitro* of chicken erythroleukemia cells transformed by temperature-sensitive mutants of avian erythroblastosis virus. *Cell* **28**, 907.

BONNER, W.M. & LASKEY, R.A. (1974). A film detection method for tritium-labelled proteins and nucleic acids in polyacryamide gels. *Eur. J. Biochem.*, **46**, 83.

CHIRGWIN, J.M., PRYZYBYLA, A.E., MACDONALD, R.J. & RUTTER, W.J. (1979). Isolation of biologically-active ribonucleic acid from sources enriched in ribonuclease. *Biochemistry*, **18**, 5294.

CLEVELAND, D.W., LOPATA, M.A., MACDONALD, R.J., COWAN, N.J., RUTTER, W.J. & KIRSCHNER, M.W. (1980). Number and evolutionary conservation of $\alpha$- and $\beta$-tubulin and cytoplasmic $\beta$- and $\gamma$-actin genes using specific cloned cDNA probes. *Cell*, **20**, 95.

DENG, G. & WU, R. (1981). An improved procedure for utilising terminal transferase to add homopolymers to the 3′-termini of DNA. *Nucl. Acids Res.*, **9**, 4173.

DESCHNER, E.E. (1983). Adenomas: pre-neoplastic events, growth and development in man and experimental systems. *Path. Annual*, **18** (1), 205.

DOWNWARD, J., YARDEN, Y., MAYES, E. & 6 others (1984). Close similarity of epidermal growth factor receptor and v-erb-B oncogene protein sequences. *Nature*, **307**, 521.

ENTERLINE, H.T. (1976). Polyps and cancer of the large bowel. In: *Current Topics in Pathology* Morson, B.C., (ed.), Vol. 63, Springer-Verlag, Berlin.

FILIPE, M.I. & BRANFOOT, A.C. (1974). Abnormal patterns of mucous secretion in apparently normal mucosa of large intestine with carcinoma. *Cancer*, **34**, 282.

FORSTER, S.J., TALBOT, I.C. & CRITCHLEY, D.R. (1984). Laminin and fibronectin in rectal adenocarcinoma: relationship to tumour grade, stage and metastasis. *Br. J. Cancer*, **50**, 51.

FRIEDMAN, E., THOR, A., HAND, P.H. & SCHLOM, J. (1985). Surface expression of tumor-associated antigens in primary cultured human colonic epithelial cells from carcinomas, benign tumors, and normal tissues. *Cancer Res.*, **45**, 5648.

GABBERT, H. & HOHN, P. (1980). Grades of atypia in tubular and villous adenomas of the human colon. An electron microscopic study. *Virch. Arch.* (*Cell Path.*), **33**, 1.

GRUNSTEIN, M. & HOGNESS, D. (1975). Colony hybridisation: a method for the isolation of cloned DNAs that contain a specific gene. *Proc. Natl Acad. Sci. USA*, **72**, 3961.

HANAHAN, D. (1983). Studies on transformation of *E. coli* with plasmids. *J. Mol. Biol.*, **166**, 557.

JASS, J.R., STRUDLEY, I. & FALUDY, J. (1984). Histochemistry of epithelial metaplasia and dysplasia in human stomach and colorectum. *Scand. J. Gastroenterol.*, **19**, 109.

KLEIN, G. & KLEIN, E. (1985). Evolution of tumours and the impact of molecular oncology. *Nature*, **315**, 190.

KONISHI, F. & MORSON, B.C. (1982). Pathology of colorectal adenomas: a colonoscopic survey. *J. Clin. Path.*, **35**, 830.

MANIATIS, T., FRITSCH, E.F. & SAMBROOK, J. (1982). Molecular Cloning: A Laboratory Manual. Cold Spring Harbor Laboratory, New York.

McMASTER, G.K. & CARMICHAEL, G.G. (1977). Analysis of single- and double-stranded nucleic acids on polyacrylamide and agarose gels by using glyoxal and acridine orange. *Proc. Natl Acad. Sci. USA*, **74**, 4835.

MUTO, T., BUSSEY, H.J.R. & MORSON, B.C. (1975). The evolution of cancer of the colon and rectum. *Cancer*, **36**, 2251.

OKAYAMA, H. & BERG, P. (1982). High efficiency cloning of full-length cDNA. *Mol. Cell Biol.*, **2**, 161.

PELHAM, H.R.B. & JACKSON, R.J. (1976). An efficient mRNA-dependent translation system from reticulocyte lysates. *Eur. J. Biochem.*, **67**, 247.

PINTO, M., APPAY, M.-D., SIMON-ASSMANN, P. & 4 others (1982). Enterocytic differentiation of cultured human colon cancer cells by replacement of glucose by galactose in the medium. *Biol. Cell*, **44**, 193.

RIGBY, P.W.J., DIECKMANN, M., RHODES, C. & BERG, P. (1977). Labelling deoxyribonucleic acid to high specific activity *in vitro* by nick-translation with DNA polymerase I. *J. Mol. Biol.*, **113**, 237.

ROTHBERG, P.G., SPANDORFER, J.M., ERISMAN, M.D., STAROSCIK, R.N., SEARS, H.R., PETERSON, R.O. & ASTRIN, S.M. (1985). Evidence that c-*myc* expression defines two genetically distinct forms of colorectal adenocarcinoma. *Br. J. Cancer.*, **52**, 629.

SCOTT, M.R.D., WESTPHAL, K.-H. & RIGBY, P.W.J. (1983). Activation of mouse genes in transformed cells. *Cell*, **34**, 557.

SHEAHAN, D.G. (1980). Dysplasia: a pathologist's view of its general applicability. In *Progress in Cancer Research and Therapy: Colorectal Cancer Prevention, Epidemiology and Screening*, Winawer, S., *et al.* (eds.). Vol. 13, p. 335. Raven Press, New York.

STEWART, J., EVAN, G., WATSON, J. & SIKORA, K. (1986). Detection of the c-*myc* oncogene product in colonic polyps and carcinomas. *Br. J. Cancer*, **53**, 1.

THOMAS, P.F. (1980). Hybridization of denatured RNA and small DNA fragments transferred to nitrocellulose. *Proc. Natl Acad. Sci. USA*, **77**, 5201.

THOMAS, P. (1983). Hybridisation of denatured RNA, transferred or dotted onto nitrocellulose paper. In *Methods in Enzymology*, Wu, R., *et al.* (eds.). Vol. 100B, p. 255. Academic Press, New York.

VEALE, A.M.O. (1965). *Intestinal polyposis*. Eugenics laboratory Memoirs, Series 40. Cambridge University Press, London.

WATSON, C.J. & JACKSON, J.F. (1985). An alternative procedure for the synthesis of double-stranded cDNA for cloning in phage and plasmid vectors. In *DNA Cloning: A Practical Approach*, Glover, D.M. (ed.). Vol. 1, IRL Press, Oxford.

WICKENS, M.P. BUELL, G.N. & SCHIMKE, R.T. (1978). Synthesis of double-stranded DNA complementary to lysozyme, ovomucoid and ovalbumin mRNAs. *J. Biol. Chem.*, **253**, 2483.

WILLIAMS, G.T. (1985). Commentary: transitional mucosa of the large intestine. *Histopathology*, **9**, 1237.

WILLIAMS, J.G. (1981). The preparation and screening of a cDNA clone bank. In *Genetic Engineering*, Williamson, R. (ed.). Vol. 1, p. 1. Academic Press, London.